# Therapeutic Approach to Primary Tic Disorders and Associated Psychiatric Comorbidities

**DOI:** 10.3390/brainsci14121231

**Published:** 2024-12-07

**Authors:** Irene Berzosa-Gonzalez, Saul Martinez-Horta, Jesus Pérez-Pérez, Jaime Kulisevsky, Javier Pagonabarraga

**Affiliations:** 1 Movement Disorder Unit, Neurology Department, Hospital de Sant Pau, 08041 Barcelona, Spain; iberzosa@santpau.cat (I.B.-G.); jperezp@santpau.cat (J.P.-P.); jpagonabarraga@santpau.cat (J.P.); 2 Department of Medicine, Universitat Autonoma de Barcelona (UAB), 08193 Barcelona, Spain; 3 Centro de Investigación Biomédica en Red-Enfermedades Neurodegenerativas (CIBERNED), 28029 Madrid, Spain; 4 Sant Pau Biomedical Research Institute (IIB-Sant Pau), 08041 Barcelona, Spain

**Keywords:** Tourette syndrome, tics, treatment, psychiatric comorbidities, clonazepam

## Abstract

Background/Objectives: The treatment of tics and psychiatric comorbidities is crucial when they affect the patient’s well-being and relationships. However, the optimal pharmacological treatment (PT) tailored to each patient’s phenotype remains unclear. The primary objective of this study is to describe the clinical characteristics and treatment received for tics and psychiatric comorbidities in our cohort of children and adult patients with tic disorders. Additionally, a further aim was to quantify the severity of tics, comorbidities and overall severity, and the overall clinical changes observed during the follow-up. Methods: Retrospective descriptive study of patients with tic disorders under follow-up at our Tic Functional Unit from January 2022 to March 2024. Two independent neurologists retrospectively applied the Clinical Global Impression of Change (CGI-C) and the Clinical Global Impression of Severity (CGI-S) scales at baseline and at last assessment. Results: A total of 36 individuals were included (63.8% males, median age = 18 years, IQR 19): 94.4% with Tourette syndrome (TS), 2.8% with chronic tic disorder (CTD), and 2.8% with provisional tic disorder (PTD). A total of 86% had at least one psychiatric comorbidity, the most common being obsessive–compulsive symptomatology (OCS) (52%), anxiety (52%), and attention deficit hyperactivity disorder (ADHD) (35%). At last assessment, 26 patients (72.2%) were on undergoing PT for tics and 3 were receiving additional botulinum toxin. The most used medication for tics were aripiprazole (46.2%) and clonazepam (46.2%), and for psychiatric comorbidities, SSRIs (42.9%), methylphenidate (19%), and benzodiazepines (57.1%). Overall improvement according to the CGI-C scale was mild (CGI-C 3). Children and adolescents showed greater improvement than adults (CGI-C 2 vs. 3; *p* = 0.005). Aripiprazole and clonazepam produced similar outcomes in reducing CGI-C. Conclusions: We observed a favorable clinical course in patients treated with aripiprazole and clonazepam, which appear to be better than that obtained with other treatments. We consider that clonazepam may be useful as a first-line monotherapy and as an adjuvant for both tics and comorbidities in selected cases.

## 1. Introduction

Tourette syndrome (TS) and chronic tic disorders (CTDs) are childhood-onset neurodevelopmental disorders characterized by sudden, repetitive, and involuntary movements (motor tics) and/or vocalizations (phonic tics) persisting for over one year, often with a fluctuating course. In provisional tic disorders (PTDs), tics last less than one year (DSM-5) [1].

The neuropathological basis of primary tic disorders remains unclear, but a multifactorial etiology is proposed, involving genetic, environmental, perinatal, and autoimmune factors. These may disrupt fronto-striatal cortico-subcortical circuits, implicating structures including the cingulate cortex, supplementary motor area, insula, putamen, and thalamus [2,3,4,5].

In TS, tics typically emerge between ages 4 and 8, peak in severity at 10–12 years, and significantly diminish in over half of patients by late adolescence. However, a subset experiences persistent, severe tics into adulthood, which are often resistant to multiple treatments and associated with increased risk of hospitalizations and disability [6].

Tics, particularly those of high intensity, frequency, or complexity, can substantially impact quality of life, compromising physical and emotional well-being, and academic/work and social relationships [7]. Additionally, they may lead to stigma and elevate the risk of suicide in this population [8,9]. Frequently, associated neuropsychiatric comorbidities, such as obsessive–compulsive disorder (OCD), attention deficit hyperactivity disorder (ADHD), and anxiety [10], are the main contributors to diminished quality of life [9,11].

Given the clinical heterogeneity of tic disorders, individualized therapeutic approaches are essential. These should consider patient preferences, the availability of therapies, and potential adverse effects [2,12].

Behavioral therapies (BTs) are recommended as first-line interventions for tics disorders in both children and adults, according to various international guidelines [13,14,15]. Among these, the Comprehensive Behavioral Intervention for Tics (CBIT) is endorsed by the American Academy of Neurology as the only therapy with high confidence evidence of efficacy [15]. CBIT has demonstrated efficacy comparable to pharmacological treatment (PT) in at least one comparative study [16].

PT is recommended when BT is unavailable, ineffective, or when severe tics require urgent management. In the United States, only haloperidol (approved in 1969), pimozide (1984), and aripiprazole (2014), are approved for tic control, while in Europe, haloperidol remains the sole approved drug [17]. Beyond antipsychotics, adrenergic agents and antiepileptics are also used [18]. These treatments are guided by clinical practice guidelines and supported by limited evidence from small randomized clinical trials, meta-analyses and case reports [17,19,20].

Second-line treatments, including botulinum toxin injections for selected tics [15] and deep brain stimulation (DBS) of the internal globus pallidus or thalamus [12,21,22], may be considered in refractory cases.

In our country, BT application in routine clinical practice is limited due to insufficient availability in public health services and a shortage of specialized professionals. PT is therefore our main strategy for managing both tics and the psychiatric comorbidities that exacerbate them. However, managing tic disorders remains challenging due to their fluctuating and multifaceted nature. Current pharmacological options often have limited efficacy, particularly in severe cases, necessitating the use of drug combinations. However, adverse effects frequently constrain their utility. Finally, no standardized protocol currently exists to guide the sequencing of drug introduction, optimal dosages, treatment duration, or appropriate drug combinations for patients with comorbidities beyond ADHD or OCD, or in refractory cases. Comparative studies are needed to evaluate the relative efficacy of available drugs [23].

The primary objective of this study is to analyze the treatment received by our cohort of patients with primary tic disorders and compare these with the management of tic control and psychiatric comorbidities described in the literature. We aim to detail the treatments used, considering demographic factors (e.g., age, sex), tic severity, and associated psychiatric comorbidities, in a series with a long follow-up period, in order to provide a greater understanding and contribute to improving the management of these complex disorders.

## 2. Materials and Methods

We reviewed a total of 45 medical records of patients under follow-up at the Tic Functional Unit (TFU) of the Movement Disorders Unit (MDU) at Hospital Sant Pau, Barcelona, from January 2022 to March 2024. We finally included 36 patients presenting with a primary tic disorder (TS, CTD, or PTD) according to DSM-5 diagnostic criteria. We excluded patients with TS treated with deep brain stimulation (*n* = 3), since it could be a confounding factor when assessing drug response and clinical evolution. We also excluded patients with secondary tic disorders (*n* = 6) such as neurodevelopmental disorders (e.g., intellectual disability, autism spectrum disorders, and genetic and chromosomal abnormalities) and tics of functional origin. We did not exclude any patient due to sex, social reason, race, family background, or other reasons.

Sociodemographic and clinical data for each patient were obtained from each patient’s medical record collected during TFU visits and recorded in our center’s electronic database. Clinical records in electronic data system were present for all patients, and we had access to all the visits performed for every patient from first visit in our center until last available visit. Therefore, we did not have missing data beyond the absence of specific reports on the response to medications at some visits during the follow-up. Two independent neurologists retrospectively administered the Clinical Global Impression of Severity (CGI-S) scale at the first and last visit of each patient to quantify the severity of tics, psychiatric comorbidities and overall severity. The Clinical Global Impression of Change (CGI-C) scale was also administered to quantify global clinical change during follow-up.

These data were anonymized and transferred to the REDCap application and subsequently analyzed using SPSS for statistical analysis.

An exhaustive search of studies published between 2000 and 2023 was carried out in the PubMed electronic databases. Search terms included ‘Tourette syndrome’, ‘tics’, ‘psychiatric comorbidities’, ‘treatment’, and their combinations using the Boolean operators AND and OR. We included studies that were clinical trials, systematic reviews, clinical practice guidelines, or observational studies published in English and Spanish between 2007 and 2023.

### Statistical Analysis

IBM SPSS Statistics version 25 software was used for statistical analysis. Continuous variables are expressed as mean ± standard deviation (SD) or as median and interquartile range (IQR, p75–p25). *t*-tests or Mann–Whitney tests were used to perform mean or median comparisons and Chi-square tests were used to compare qualitative variables, with significance set at *p* ≤ 0.05. Categorical variables are expressed as frequencies and percentages. The population size available for unicentric studies on TS is inherently limited, which impacts the sample size and constrains the ability to perform a robust power analysis. Nevertheless, this study included more than 30 patients, with the clinical features of the sample following a normal distribution. This enabled a thorough descriptive analysis of the clinical data and facilitated univariate comparisons between groups.

## 3. Results

### 3.1. Demographic Data

We included a total of 36 patients with tic disorders. Males accounted for 23/36 (63.8%) of the patients, with a male-to-female ratio of 2:1. The majority of patients were non-Hispanic White (97.2%) and one patient was Asian. The median age of the cohort was 18 years (IQR 19), ranging from 9 to 60 years, and significantly differed between females (24 years, IQR 30) and males (16 years, IQR 16) (*p* = 0.031). A total of 17 out of 36 individuals (47.2%) were children and adolescents (<18 years old) and 19/36 (52.8%) were adults.

### 3.2. Tic Disorders

A total of 34 of 36 patients (94.4%) received a TS diagnosis, 1 (2.8%) received a CTD diagnosis, and 1 (2.8%) received a PTD diagnosis. The median age of tic onset was 5 years (range 2 to 22 years, IQR 3, 3 values missing), with no differences between sexes (*p* = 0.686). Median age at the first consultation in our TFU/MDU was 15.5 years (range 6 to 49 years, IQR 16.5), with no differences between sexes (*p* = 0.115). Out of the 36 patients, 19 (52.8%) were followed up using neurology; 9 (25%) using neurology and psychiatry; 6 (16.7%) using neurology, psychiatry, and psychology; and 2 patients (5.6%) using neurology and psychology. A total of 31 patients were visited more than once in the TFU/MDU: 23 patients were followed up for less than 5 years, 5 patients between 5 and 10 years, and 3 patients for more than 10 years. Female patients had a longer follow-up period (*p* = 0.022) (Table 1).

### 3.3. Psychiatric Comorbidities

Patients with a “pure” tic disorder accounted for 8.3%, while 91.7% had experienced at least one psychiatric comorbidity in their lifetime. All female patients had experienced at least one comorbidity compared to 87% of males (*p* = 0.537). At last assessment, 31/36 (86.1%) had some psychiatric comorbidity, namely 12/31 (38.7%) female patients compared with 19/31 (61.3%) male patients, all of whom met criteria for TS. The patient with PTD had not experienced any psychiatric comorbidity in their lifetime, while the patient with CTD had previously exhibited obsessive traits and a depressive adjustment disorder. Among patients with psychiatric comorbidities, 11/31 (35.5%) had only one comorbidity while 20/31 (64.5%) had two or more (see Table S1, available online). The most prevalent disorders were obsessive–compulsive symptomatology (OCS) in 16/31 (52%) patients, anxiety in 16/31 (52%), ADHD in 11/31 (35%), affective disorder in 6/31 (19%), and behavioral disorder in 4/31 (13%). Anxiety and OCS were more prevalent in women than in men (*p* = 0.003) (*p* = 0.024), and ADHD was more frequent in men (*p* = 0.025) (Table 2).

### 3.4. Treatment of Tics

In our sample, only two patients (5.6%) had never received any type of treatment for tic control, while the majority (94.4%) required PT: 25/36 (69.4%) patients underwent PT only; seven patients (19.4%) took medication along with botulinum toxin injections; one patient (2.8%) underwent both PT and BT; and one patient (2.8%) received neurofeedback in addition to medication. At the last assessment, 26 patients (72.2%) were undergoing PT, 3 of whom also received botulinum toxin injections along with medication. None of them were receiving BT for tics.

#### 3.4.1. Pharmacological Treatment

**Figure 1:**
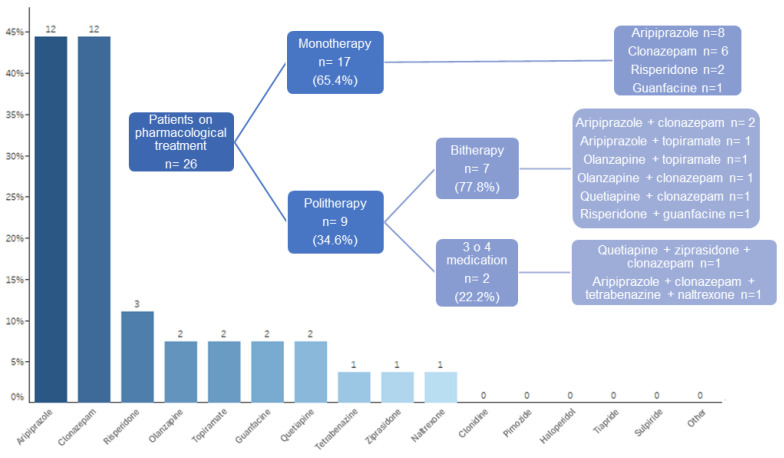
Medication and combinations used for tics at last assessment: percentage and number of cases per medication/s.

At last assessment, the two most commonly used medications were aripiprazole in 12 patients (46.2%) and clonazepam in 12 patients (46.2%), followed by other atypical antipsychotics and medications in smaller proportions (Figure 1). A total of 12 out of 17 minors and 14 out of 19 adults were on PT. The frequency of use in each age group is documented in Table S2, available online. No patient was undergoing treatment with tiapride, sulpiride, haloperidol, pimozide, clozapine, clonidine, lurasidone, or other medications. The proportion of patients on monotherapy or polytherapy and the type of medication used are reflected in Figure 1.

The average doses used for each medication for tic control are shown in Table 3.

Throughout their lives, 34 patients in the cohort had tried PT for tics; on average, each patient had tried three different medications, ranging from one to twelve different medications (IQR 4).

PT received in the past for tic control and the reasons for discontinuation are indicated in Table S3, available online.

The reported side effects of the different medications prescribed for tic control are recorded in Table S4, available online.

#### 3.4.2. Botulinum Toxin Treatment

At the last assessment, three patients were receiving botulinum toxin treatment in addition to medication. Table S5, available online, reflects the types of tics, the infiltrated muscles, the doses used, and the response.

#### 3.4.3. Treatment of Psychiatric Comorbidities

At the last assessment, 31 patients had at least one psychiatric comorbidity. Among them, 21 (67.7%) were receiving PT for these conditions; 11/21 (52.4%) were undergoing monotherapy, and 10/21 (47.6%) were taking two or more medications (see Table S6, available online). The most commonly used medications for treating psychiatric comorbidities were as follows: selective serotonin reuptake inhibitors (SSRIs) 9/21 (42.9%), tricyclic antidepressants 1/21 (4.8%), atypical antidepressants 1/21 (9.5%), benzodiazepines 12/21 (57.1%), antiepileptics 3/21 (14.3%), methylphenidate 4/21 (19%), and atomoxetine 1/21 (4.8%). No patient was taking serotonin–norepinephrine reuptake inhibitors (SNRIs) or norepinephrine–dopamine reuptake inhibitors (NDRIs). Additionally, at least 11/31 (35.5%) patients had received some kind of BT for anxiety and/or OCS throughout their lives, this consisted mainly of psychotherapy and one case of exposure and response prevention.

### 3.5. Indicators of Severity and Response to Medication

The global severity, tic severity, and psychiatric comorbidities were assessed in the 36 cases at the first and last visits with the CGI-S scale. At first visit, we observed a global severity of 4 (moderately ill), with tic severity being slightly higher compared to psychiatric comorbidities. By age group, the severity of comorbidities in adults was significantly higher than in children/adolescents (*p* = 0.042). The severity of tics and psychiatric comorbidities were similar between men and women and across age groups (*p* > 0.10 in all comparisons) (see Table S7, available online). At the last visit, the overall severity was 3 (mildly ill), with psychiatric comorbidities being slightly worse with respect to tics. By age group, adults had significantly greater severity than children/adolescents both in terms of tics (*p* = 0.038) and psychiatric comorbidities (*p* = 0.002). No statistically significant differences were found according to sex (*p* > 0.10 in all comparisons) (see Table S8, available online).

**Figure 2:**
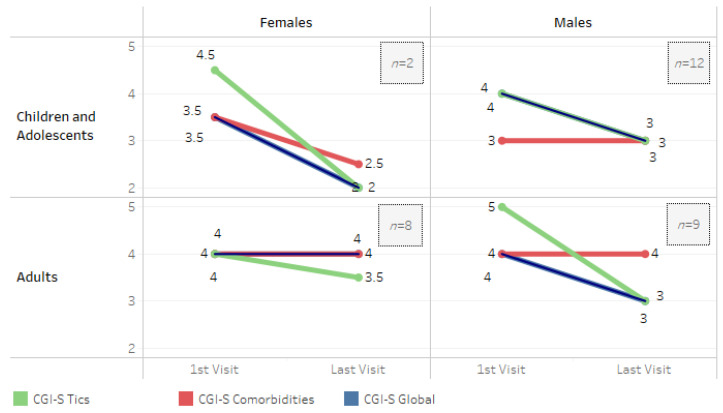
CGI-S according to age group and sex. 1 = normal; 2 = borderline ill; 3 = mildly ill; 4 = moderately ill; 5 = markedly ill; 6 = severely ill; 7 = among the most extremely ill patients.

**Figure 3:**
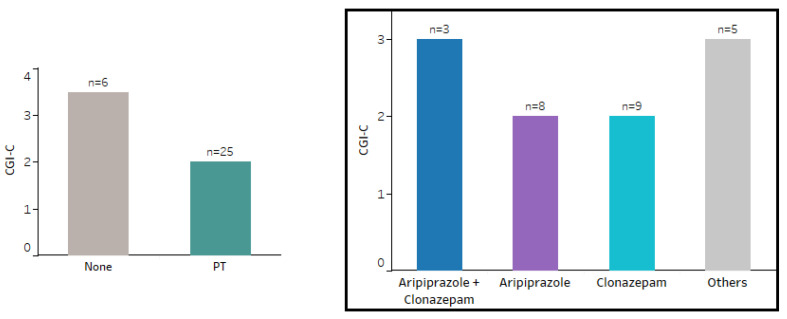
CGI-C in patients with pharmacotherapy (PT) vs. without PT (**left**) and CGI-C according to pharmacological group (**right**). 1 = very much improved; 2 = much improved; 3 = minimally improved; 4 = no change; 5 = minimally worse.

The CGI-C scale was assessed in 31 patients who were seen more than once in the TFU/MDU, 25 of whom were receiving PT, while 6 were not. On average, patients minimally improved (CGI-C 3) throughout the follow-up period, and children/adolescents improved more than adults (CGI-C 2 vs. 3) (*p* = 0.005). No statistically significant differences were found according to sex (*p* = 0.755). Adult women appeared to exhibit a more persistent and severe course compared to the other groups. However, statistical comparison was not feasible due to the limited number of patients in each group (Figure 2). Patients undergoing PT showed more favorable outcomes compared to those who did not receive treatment, as evidenced by a higher proportion of patients achieving a CGI-C score of 2 at follow-up. However, statistical comparison was not feasible due to the limited number of patients without PT (Figure 3).

Patients undergoing PT showed a trend toward a more favorable outcome compared to those not receiving treatment, although this difference did not reach statistical significance (CGI-C 2 vs. 3, *p* = 0.050) (Figure 3).

Patients in the aripiprazole and clonazepam treatment groups achieved comparable outcomes in reducing CGI-C scores, with similar proportions reaching a CGI-C score of 2. In contrast, a higher frequency of CGI-C scores of 3 was observed with other treatments or treatment combinations (Figure 3).

## 4. Discussion

### 4.1. Demographic Data

The clinical expression of tic disorders, particularly TS, varies according to age and sex. During childhood, these conditions are more prevalent in males than females, with a ratio of 4:1. This disparity may be partially explained by the increased detection of tics when assessing concurrent psychiatric comorbidities (e.g., ADHD, behavior disorders), and the greater severity of tics in boys compared to girls [24]. However, in adulthood, women often experience a more persistent and severe course, leading to a greater impact on their quality of life [24,25,26].

In our cohort, 63.8% of the patients were male. This unequal sex distribution in the inclusion of patients reflects the overall higher prevalence of the condition in males. Age analysis revealed a similar pattern, with a higher proportion of boys than girls (ratio 4:1) during childhood, whereas in adulthood, the sex distribution became balanced (1:1). Consistent with previous studies [27,28,29], adult women in our cohort seem to have a more unfavorable clinical course compared to the other groups. This might explain why females were older (*p* = 0.031) and had a longer follow-up period than males (*p* = 0.022).

### 4.2. Tic Disorders

The majority of patients had a diagnosis of TS (94.4%), with a minority of patients having CTD (2.8%) and PTD (2.8%). The latter two are more prevalent in the general population, at up to 25%, and school-age children, at 1–2%, respectively. Although TS has a prevalence of around 1% [30], it is often associated more with neuropsychiatric disorders [2], which are frequently the main reason for seeking medical advice.

The median age of tic onset in our study was 5 years, with no differences by sex, which is consistent with findings in the literature [2,12]. At the first visit to our MDU/TFU, the median age of patients was 15 years, with a wide range of ages from 6 to 49. The tendency towards older ages at the first visit could be explained by the fact that patients first seen in the TFU may be referred from other centers in Catalonia after being evaluated by different specialists throughout their lives.

### 4.3. Psychiatric Comorbidities

Our patient sample aligns with a cross-sectional study of 1374 patients, suggesting that “pure” TS is an exception; similarly, we found that up to 91% of patients with TS have received at least one psychiatric comorbidity diagnosis in their lifetime, with more than half diagnosed with two or more [10].

It is noteworthy that all our female patients had at least one comorbidity in the past. At the last assessment, all patients with psychiatric comorbidities were diagnosed with TS.

Consistent with other studies [7,10], we found a high prevalence of OCS (52%), anxiety (52%), and ADHD (35%). To a lesser extent, we also found affective disorders (19%) and conduct disorders (13%). Anxiety and OCS were significantly more prevalent in females (*p* = 0.003 and *p* = 0.024), while ADHD was more prevalent in males (*p* = 0.025), as reported in some studies [25,27].

The most observed comorbidity association in our sample is between anxiety and OCS in 6/31 patients. There is also a tendency for co-occurring conduct/impulsivity disorders in patients with ADHD and obsessive–compulsive traits. The phenotypes of ADHD combined with conduct disorder, as well as affective-anxious symptomatology paired with OCD, have been thoroughly explored in previous research [31].

### 4.4. Treatment of Tics

#### 4.4.1. Behavioral Treatment

Although BT is considered the treatment of choice for mild tics by various international guidelines [14], in our sample, only one child had received BT for tics and anxiety management. This was due to the unavailability of BT for tics in our center and surrounding areas, as well as a shortage of specialized healthcare professionals in our national public health system.

However, it is important to note that all patients and family members/companions seen in the FTU received psychoeducation about tic disorders from neurologists and/or neuropsychologists from the first visit, aiming to enhance the understanding of and empowerment relating to their condition.

#### 4.4.2. Pharmacological Treatment

The majority of patients (94.4%) had tried some form of PT. At the last assessment, 26 patients (72.2%) were on PT, with 65.4% of them receiving monotherapy and 34.6% combining two or more medications. By age, treatment was received by 70.6% of children/adolescents and 73.7% of adults. By sex, there was a greater tendency towards medication use in females compared to males for tic control (76.9% vs. 69.6%) and also for psychiatric comorbidities (76.9% vs. 61.5%), although the observed difference was not significant. This might suggest a more unfavorable phenotype in females needing medical treatment.

The most commonly used medications in our cohort were aripiprazole and clonazepam, in equal proportion (46.2%). Aripiprazole was administered to a greater extent as a monotherapy (66.7%), while clonazepam was prescribed in half of the cases as monotherapy and in the other half in combination with one or more drugs. Risperidone follows as the next most frequent (11.5%), being used twice in monotherapy and once in dual therapy; other atypical antipsychotics (olanzapine, quetiapine, ziprasidone), topiramate, guanfacine, and, less frequently, tetrabenazine and naltrexone, were also used to a similar extent, with the latter taken by a patient who also had alcohol dependence.

If we observe the use between children/adolescents and adults, the most prescribed medication for the former in the last assessment was clonazepam (58.3%), closely followed by aripiprazole (41.7%), then risperidone (8.3%) and guanfacine (8.3%). However, among adults, there was a somewhat greater preference for aripiprazole (50%) versus clonazepam (35.7%). Furthermore, the additional use of drugs other than guanfacine and risperidone was more widespread than in children.

The latest European guideline for TS by the European Society for the Study of Tourette Syndrome (ESSTS) in 2022, based on a survey of 59 clinical experts, captured treatment preferences for TS in clinical practice, also distinguishing between children/adolescents and adults. Overall, there was a high preference for aripiprazole in both age groups, which was consistent with our results. The biggest difference between groups was the use of haloperidol, the second most preferred in adults, while in children/adolescents, tiapride, clonidine, and guanfacine were mentioned more frequently, as these are recommended for mild–moderate tics in concurrent ADHD. Following these, risperidone was recommended with good clinical evidence but with more limited use due to metabolic effects and weight gain [17].

However, in our cohort, no patient received treatment with tiapride, haloperidol, clonidine, pimozide, amisulpride, or sulpiride. Although haloperidol is the only drug approved in Europe for tic treatment, with well-established efficacy and experience, its clinical use is currently restricted to selected cases of severe, treatment-refractory tics due to the increased likelihood of undesirable adverse effects, such as extrapyramidal symptoms [15,17]. This may explain why no patients were treated with haloperidol at the latest assessment in our study. Table S3 in the Supplementary Materials shows that eight patients received haloperidol in the past, but the treatment was discontinued for various reasons.

It is noteworthy that clonazepam is not considered a common treatment for TS either according to the ESSTS medication list or the American guidelines [15], while in our study, it ranked as the first and second most frequently used drug in both children/adolescents and adults. It is a medication that has been used in the past for TS [32] and is currently considered third-line therapy [33]. Single-blind studies have shown efficacy equal to or greater than clonidine or haloperidol [13], although controlled studies are needed.

In our experience, clonazepam may be effective as first-line monotherapy for milder cases, especially when there is concurrent anxiety or sleep disorders, with the possibility of slow progressive titration. It can also be effective as an adjunct when an expected response is not achieved with other drugs that may cause unwanted effects at high doses.

As additional information, we provide our combinations of tic medications and associated psychiatric comorbidities. In the literature review, we found no recommendations regarding the experience with drug combinations in tics and indications according to associated comorbidities, beyond recommendations of risperidone and/or aripiprazole in addition to SSRIs and BT in patients with OCS and adrenergic agents for ADHD/impulsivity [17]. It would be interesting for future guidelines to also reflect the experience and evidence of these drug combinations.

The average doses used for each medication were within the ranges suggested by various guidelines [28,34].

Overall, the reason for drug withdrawal for tics was due to inefficacy and adverse effects but also to avoid medium- to long-term adverse effects and, in very few cases, due to clinical improvement. Among the drugs discontinued in the past, typical antipsychotics, such as pimozide in 15 patients and haloperidol in 8 patients, stood out. Clonazepam was also tried in 12 patients and aripiprazole in 12 patients, with the same proportion abandoned due to inefficacy or adverse effects. Of these, aripiprazole was reintroduced in two patients and clonazepam in one. We consider it worthwhile to retry a medication that has been ineffective or has produced adverse effects in the past, given the fluctuating nature of the disease, the weight of comorbidities in tic exacerbation at each moment and the maturation of neuronal networks and the individual in general over time.

Most of the adverse effects recorded in our cohort have been described as frequent or very frequent in the literature. Some patients experienced different adverse effects from the same treatment. Olanzapine and topiramate caused more adverse effects proportionally. We did not observe any serious adverse events, such as cardiac problems due to QT prolongation or extrapyramidal symptoms. This could be due to the increasing use of atypical antipsychotics with a better safety profile such as aripiprazole and the use of minimal necessary doses.

#### 4.4.3. Botulinum Toxin Treatment

According to the AAN guidelines for TS, the local administration of botulinum toxin is likely more effective than placebo in reducing tics, as well as associated premonitory sensations [15]. In our cohort, 7 out of 36 patients had been injected with botulinum toxin for motor tic control at some point in their lives and 3 continued this treatment at the last assessment. Those were young adults with insufficient response to tic control drugs, and in one case, due to patient reluctance to try some medication for personal reasons.

Our experience suggests that toxin therapy may be effective for treating focal ocular motor tics (using dosages and distribution similar to those for blepharospasm), as well as for alleviating certain shoulder and foot tension tics and associated premonitory sensations. However, it appears to be less effective in controlling arm tics.

### 4.5. Treatment of Psychiatric Comorbidities

The most commonly used treatment in our cohort were SSRIs in patients with OCS, anxiety, and depressive disorders, with methylphenidate and atomoxetine used for ADHD and clonazepam prescribed for various conditions, especially anxiety.

Furthermore, eleven patients received BT for psychiatric comorbidities, mainly psychotherapy for anxiety, and one patient underwent exposure and response prevention for OCD.

Our study highlights disparities in psychological assessment and follow-up. Access to psychological care in our setting is influenced by the availability and accessibility of services, which depend on both the resources allocated by the public health system and patients’ financial ability to afford private alternatives.

However, we consider that the specific management and follow-up of psychiatric comorbidities in patients with tic disorders should be conducted in collaboration with the psychiatry and psychology teams. It is essential to evaluate the feasibility of administering BT, as its implementation can significantly contribute to improving both psychiatric comorbidities and tic control.

### 4.6. Severity Indicators and Drug Response

The overall severity of the cohort at the first visit was moderate. Tics had a slightly greater impact than psychiatric comorbidities. The latter affected adults more than children/adolescents (*p* = 0.042), with no significant differences between sexes (*p* = 0.75).

During the follow-up period, we observed a slight overall improvement (CGI-C of 3, CGI-S from moderate to mild); this was greater in tics (1.1 point reduction in CGI-S) than in psychiatric comorbidities (0.3 CGI-S points).

Children/adolescents improved more than adults (CGI-C 2 vs. 3, *p* = 0.005), in line with the most commonly expected course, in which tics usually decrease as one moves towards adolescence and adulthood [28]. Adult women appeared to have a more persistent and severe course compared to the other groups (Figure 2), as other studies have described previously [25].

These results emphasize the need for a more comprehensive approach to psychiatric comorbidities, not only in the adult and female populations, which appear to be more affected but also in children/adolescents, since the presence of comorbidities in childhood has been identified as a potential poor prognostic factor in adulthood [35,36].

As expected, patients undergoing PT appeared to have more favorable outcomes compared to those without treatment.

To date, there is a lack of head-to-head studies to help us understand which medications are most effective for controlling tics [23]. In our sample, patients in the aripiprazole and clonazepam treatment groups achieved comparable outcomes in reducing CGI-C scores, with similar proportions reaching a CGI-C score of 2. In contrast, a higher frequency of CGI-C scores of 3 was observed with other treatments or treatment combinations.

### 4.7. Limitations and Strengths

This study has several limitations. The primary limitation is the small sample size, which is due to the rarity of TS and the single-center study design. Additionally, the study was conducted in a specialized clinical setting, which could introduce bias by capturing only the subset of patients who seek specialized care, often those with more severe symptoms. This selection bias may affect the generalizability of the findings. The small sample size and selection bias may also impact the external validity and statistical power of the results, highlighting the need for further validation in larger, more diverse cohorts. Another limitation is the retrospective nature of the study, which restricted the data to information available in the patients’ clinical histories. Practical constraints at our center prevented the use of the Yale Global Tic Severity Scale and other recognized scales. Instead, we employed the CGI-C and the CGI-S. These scales, while suitable for retrospective application based on patient records, provided a uniform assessment aligned with the study design, enabling the observation of the general evolution of the condition over the follow-up period. However, the retrospective design and chosen scales do not allow for definitive conclusions regarding the direct relationship between medications and clinical outcomes. This is compounded by the inherently non-linear nature of tic disorders and the potential influence of external factors on tic expression. Lastly, the absence of a control group due to the limited sample size further constrains the study’s conclusions.

The main strength of this study is the contribution of such a detailed description of the therapeutic approach to a sample of patients with a minority disease. At our FTU level, this study has allowed us to better understand our patients and, as a result, will allow us to optimize therapeutic management in our daily clinical practice.

### 4.8. Future Research and Practical Implications

To better establish causality, future research could implement a prospective cohort design or, ideally, a randomized controlled trial to assess the direct effects of specific medications or therapies on tic severity and quality of life, using validated and recognized scales. It would be interesting that future research focus on tailored treatment strategies for patients with specific comorbidities, such as ADHD or OCD, to enhance therapeutic outcomes. Additionally, investigations into non-pharmacologic interventions, including behavioral therapy, lifestyle modifications, and caregiver interventions, could provide valuable insights into comprehensive tic disorder management.

Given the complexity of tic disorders, a multidisciplinary approach involving neurologists, psychiatrists, and behavioral therapists may prove advantageous. Such an approach could lead to practical recommendations, such as developing guidelines for monitoring and managing medication side effects, especially for treatments with known risks. Furthermore, engaging families and caregivers in treatment plans may help ensure long-term adherence and optimize patient outcomes, emphasizing the critical role of support systems in managing these disorders.

## 5. Conclusions

Overall, our clinical experience underscores the complexity of TS and CTD, which require a multidisciplinary approach tailored to each patient. The treatment of TS remains challenging due to its fluctuating, multifaceted nature and associated comorbidities, with current pharmacological options often offering limited efficacy, especially in severe cases. In this study, pharmacological intervention was essential, as behavioral therapies were not viable. Aripiprazole, widely used in our cohort, demonstrated favorable outcomes, in agreement with available scientific evidence. Clonazepam appears to be effective both as a first-line monotherapy and as an adjunctive treatment for managing tics and comorbidities. Nonetheless, further randomized controlled trials are required to establish optimal pharmacological strategies and drug combinations tailored to individual patients.

## Figures and Tables

**Table 1:** Sociodemographic and clinical characteristics of our sample and differences by sex.

Characteristics (*n*, %)	Total 36 (100)	Males 23 (63.8)	Females 13 (36.2)	*p* Value
Tic disorder (*n*, %): - Tourette Syndrome - Chronic tic disorder - Provisional tic disorder				
	34 (94.4)	21 (91.3)	13 (100)	0.673
	1 (2.8)	1 (4.35)	0	
	1 (2.8)	1 (4.35)	0	
Median age at last assessment (IQR)	18 (19)	16 (16)	24 (30)	0.031
Median age at tic onset (IQR)	5 (3)	5 (5)	5.5 (2.25)	0.686
Median age at first visit (IQR)	15.5 (16.5)	14 (16)	20 (20)	0.115
Psychiatric comorbidities at last assessment (*n*, %)	31 (86.1)	19 (82.6)	12 (92.3)	0.634
Treatment for tics at last assessment (*n*, %)	26 (72.2)	16 (69.6)	10 (76.9)	0.721
Treatment for psychiatric comorbidities at last assessment (*n*, %)	21 (58.3)	13 (56.5)	8 (61.5)	0.649
Follow up > 1 visit FTU/MDU (*n*, %) - Median years (range) - <5 years - 5–10 years - >10 years	31 (86)	21 (91.3)	10 (76.9)	0.186 0.022
	2 (0–24)	2 (0–8)	4.5 (0–24)	
	23 (64)	18 (78.3)	5 (38.4)	
	5 (14)	3 (13)	2 (15.4)	
	3 (8)	0	3 (23.1)	
Follow up - Neurology - Neurology and Psychiatry - Neurology, Psychiatry, and Psychology - Neurology and Psychology				
	19 (52.8)			
	9 (25)			
	6 (16.7)			
	2 (5.6)			

**Table 2:** Differential distribution of psychiatric comorbidities in women and men.

	Total (*n* = 31) (%)	Females (*n* = 12) (%)	Males (*n* = 19) (%)	*p* Value
OCS	16 (52)	9 (75)	7 (37)	0.024
Obsessive–compulsive traits	9 (29)	5 (42)	4 (21)	
OCD	7 (23)	4 (33)	3 (16)	
Arithmomania/arithmophobia	4 (13)	2 (17)	2 (11)	
Anxiety	16 (52)	10 (83)	6 (32)	0.003
Anxiety disorder	15 (48)	9 (75)	6 (32)	
Social phobia	1 (3)	1 (8)	0 (0)	
ADHD	11 (35)	1 (8)	10 (53)	0.025
Affective disorder	6 (19)	2 (17)	4 (21)	0.877
Dysthymic disorder	3 (10)	2 (17)	1 (5)	
Adjustment disorder	2 (6)	0 (0)	2 (11)	
Depression	1 (3)	0 (0)	1 (5)	
Behavioral disorder	4 (13)	0 (0)	4 (21)	0.111
Oppositional defiant disorder	1 (3)	0 (0)	1 (5)	
Conduct/impulsivity disorder	3 (10)	0 (0)	3 (16)	
Autism spectrum disorder	1 (3)	1 (8)	0 (0)	0.177
Misophonia	1 (3)	1 (8)	0 (0)	0.177
Dissociative identity disorder	1 (3)	1 (8)	0 (0)	0.177
Personality disorder	1 (3)	0 (0)	1 (5)	0.446
Specific language disorder	1 (3)	0 (0)	1 (5)	0.446
Functional disorder	1 (3)	1 (8)	0	0.177
Eating disorder	1 (3)	0 (0)	1 (5)	0.446
Substance abuse disorder	1 (3)	0 (0)	1 (5)	0.446

OCS: obsessive–compulsive symptomatology; OCD: obsessive–compulsive disorder; ADHD: attention deficit hyperactivity disorder.

**Table 3:** Average dose of each medication to control tics (mg) at last assessment.

Medication	*n* = 26	Mean	SD	Minimun	Maximun
*Aripiprazole*	12	6.46	3.96	2.50	15.00
*Clonazepam*	12	1.20	0.86	0.50	3.00
*Risperidone*	3	2.17	0.29	2.00	2.50
*Olanzapine*	2	7.50	3.53	5.00	10.00
*Quetiapine*	2	25	0	25	25
*Guanfacine*	2	2.5	0.71	2	3
*Topiramate*	2	125	106.1	50	200
*Ziprasidone*	1	20	.	.	.
*Tetrabenazine*	1	75	.	.	.
*Naltrexone*	1	50	.	.	.

## Data Availability

The data that support the findings of this study are available from the corresponding author upon reasonable request due to ethical reasons.

## Supplementary Materials

The following supporting information can be downloaded at: https://www.mdpi.com/article/10.3390/brainsci14121231/s1; Table S1. Psychiatric comorbidities and associations in our cohort; Table S2. Tic treatment and usage frequency by age group at last visit; Table S3. Treatment received for tics and reason for abandoning it; Table S4. Reported adverse effects secondary to medication for tic control; Table S5. Patients receiving infiltration with botulinum toxin, infiltrated muscles, dose and effect; Table S6. Medication and medication combinations for the treatment of psychiatric comorbidities; Table S7. CGI-S at first visit according to age group and sex; Table S8. CGI-S at last visit according to age group and sex.
