# Supplementary material for: Therapeutic Approach to Primary Tic Disorders and Associated Psychiatric Comorbidities
Source: Brain Sci. 2024 Dec 7;14(12):1231. doi: 10.3390/brainsci14121231 (PMC11726857; doi:10.3390/brainsci14121231)
Supplement: Supplementary file 1 [file brainsci-14-01231-s001.zip › brainsci-3326980-supplementary.pdf]

Table S1. Psychiatric comorbidities and associations in our cohort.

**1 comorbidity, n= 11 (35.5%)**		**≥ 2 comorbidities, n= 19 (64.5%)**	
n= 5	ADHD	n=1	Anxiety + ADHD
n=3	OCS	n=1	Anxiety + personality disorder
n= 2	Anxiety	n=1	Anxiety + misophonia
n=1	Adjustment disorder with depressed mood	n=1	Anxiety + functional disorder
		n=1	Anxiety + conduct/impulsivity disorder
		n=6	Anxiety + OCS
		n=2	Anxiety + OCS + dysthymic disorder
		n=1	Anxiety + social phobia + obsessive-compulsive traits + ADHD
		n=1	OCD + ED + mixed adjustment disorder
		n=1	Depression + OCD + alcohol dependence + ADHD
		n=1	Obsessive compulsive traits + ADHD + conduct/impulsivity disorder
		n=1	ADHD + oppositional defiant disorder + SLI
		n=1	ADHD + conduct/impulsivity disorder
		n=1	Dysthymic disorder + obsessive-compulsive traits + ASD + dissociative identity disorder

ED: eating disorder; ADHD: attention deficit hyperactivity disorder; OCS: obsessive-compulsive symptomatology including obsessive compulsive disorder (OCD), obsessive-compulsive traits and arithmomania/arithmophobia; ASD: autism spectrum disorder; SLI: specific language impairment

Table S2. Tic treatment and usage frequency by age group at last visit

**Children and adolescents n= 12**		**Adults n= 14**	
n= 7 (58.3%)	Clonazepam	n= 7 (50%)	Aripiprazole
n= 5 (41.7%)	Aripiprazole	n= 5 (35.7%)	Clonazepam
n= 1 (8.3%)	Risperidone	n= 2 (14.3%)	Risperidone
n= 1 (8.3%)	Guanfacine	n= 2 (14.3%)	Olanzapine
n= 0	Olanzapine	n= 2 (14.3%)	Quetiapine
n= 0	Ziprasidone	n= 2 (14.3%)	Topiramate
n= 0	Quetiapine	n= 1 (7.1%)	Ziprasidone
n= 0	Tetrabenazine	n=1 (7.1%)	Guanfacine
n= 0	Topiramate	n= 1 (7.1%)	Tetrabenazine
n= 0	Naltrexone	n= 1 (7.1%)	Naltrexone

Table S3. Treatment received for tics and reason for abandoning it.

**Medication**	**n**	**Inefficiency**	**Adverse Event (AE)**	**Unknown**	**Other**	**Clinical improvement**
Aripiprazole	12	4	5	2	1 (patient's decision)	.
Risperidone	5	3	2	.	.	.
Olanzapine	5	.	4	.	1 (change to avoid AE)	.
Quetiapine	1	.	1	.	.	.
Ziprasidone	2	1	1	.	.	.
Pimozide	15	6	6	2	1 (change because of ADHD)	.
Tiapride	2	1	.	1	.	.
Haloperidol	8	2	1	2	2 (change to avoid AE)	1
Clonidine	3	1	2	.	.	.

Guanfacine	3	1	2	.	.	.
Clonazepam	12	3	4	2	2 (change for another, pregnancy)	1
Tetrabenazine	5	1	3	.	.	1
Topiramate	7	1	4	2	.	.
Naltrexone	3	2	1	.	.	.
Other*	2	1	1	.	.	.

*Other: P1: chlorpromazine, levomepromazine, ondansetron, lamotrigine; P2: pregabalin

Table S4. Reported adverse effects secondary to medication for tic control.

**Medication**	**Adverse events**	**Frequency according to the AEMPS**
Aripiprazole	- Drowsiness	- Frequent
	- Seizure with generalized tonic-clonic evolution	- Frequency not known
	- Akathisia	- Frequent
	- Anxiety	- Frequent
	- Nausea and vomiting	- Frequent
	- Depressive symptoms	- Uncommon
	- Muscle tension and paresthesias in the frontal region	- Not described
	- Auditory hallucinations	- Not described
	- Sensation of fleeting visions of passage	- Not described
Risperidone	- Sedation	- Very frequent
	- Discomfort	- Uncommon
	- Urination urgency	- Not described
Olanzapine	- Craving for alcohol	- Not described
	- Weight gain	- Very frequent
	- Increased appetite	- Frequent
	- Drowsiness	- Very frequent
	- Fatigue	- Frequent
Quetiapine	- Confussion	- Not described
Ziprasidone	- Insomnia	- Frecuency not known
Pimozide	- Drowsiness	- Very frequent
	- Weight gain	- Very rare
	- Erectile dysfunctionl	- Frequent
	- Decreased libido	- Very rare
	- Pseudocrisis	- Not descrbed
	- Visual hallucinations	- Not described
Clonidine	- Hypotension	- Very frequent
	- Sedation	- Very frequent
Guanfacine	- Bradycardia	- Frequent
	- Hypotension	- Frequent
Clonazepam	- Drowsiness	- Frequent
	- Fatigue	- Frequent
	- Restless leg syndrome	- Not described
	- Memory los	- Frequent
Tetrabenazine	- Akathisia	- Frecuency not known
	- Verbiage	- Not described
	- Mania	- Not described
	- Insomnia	- Frequent
	- Feeling "numb"	- Not described
	- Sedation	- Very frequent
	- Depression	- Very frequent

Topiramate	- Diplopia<br>- Sparkling scotoma<br>- Increased anxiety<br>- Increased nervousness<br>- Irritability<br>- Mental slowness	- Frequent<br>- Frequency not known<br>- Frequent<br>- Frequent<br>- Frequent<br>- Frequent
Naltrexone	- Pseudocrisis	- Not described
Other	- Lamotrigine: increased nervousness	- Frequent

Frequency described for each drug according to the AEMPS technical sheet: very common (≥1/10), common (≥1/100 to ≤1/10), infrequent (≥1/1000 to ≤1/100); rare (≥1/10000 to ≤1/1000); very rare (<1/10000); frequency not known (cannot be estimated from available data). Not described: if not included in the technical sheet. AEMPS: Spanish Agency for Medicines and Health Products

Table S5. Patients receiving infiltration with botulinum toxin (n=3), infiltrated muscles, dose and effect.

	**Tics**	**Total dose BTX**	**Infiltrated muscles and dose**	**Total number of infiltrations**	**Effect**
**1**	Complex motor tic: sudden flexion of both elbows, anterior cervical throw and shoulder abduction	OnaA: 150 U	75 U /Trapezius<br><br>(1st infiltration + biceps, long supinator, left deltoid muscle)	3	Effective for shoulder tics and associated local muscle tension but ineffective for arm tics<br><br>every 3-4 months
**2**	Tension tics right foot and right arm (fist throwing)	OnaA: 50 U	50 U right foot muscles<br><br>(1st and 2nd infiltration total of 150 U: 5 U right extensor digitorum brevis muscle, 20 U interosseous right foot, 70 U right quadriceps, 15 U biceps+ 15 lateral triceps + 15 U long supinator)	3	Effective for foot tic and tension relief, lesser effect and duration for thee control of right arm tics<br><br>every 6 months
**3**	Blinking	OnaA: 50 U	20 U c/periorbicularis + 5 U c/pretarsal	2	Good<br>every 3 months

OnaA: onabotulinum toxin A

Table S6. Medication and medication combinations for the treatment of psychiatric comorbidities

	**SSRIS**	**TCAS**	**ATYP**	**BZD**	**ANTIEPI**	**METHYL**	**ATOMX**	**COMORB**
1				X				Anxiety, obsessive-compulsive traits
**2**						X		ADHD
3	X		X		X			Anxiety, obsessive-compulsive traits
**4**			X	X				Anxiety, obsessive-compulsive traits
5	X							Anxiety, OCD

**6**						X	ADHD
**7**	X		X				Anxiety, conduct/impulsivity disorder
**8**	X						OCD, ED, arithmomania/arithmophobia, adjustment disorder with mixed anxious and depressed mood
**9**			X				Anxiety
**10**				X			Anxiety, OCD, arithmomania/arithmophobia
**11**	X		X				Adjustment disorder with depressed mood
**12**	X		X				Anxiety, OCD
**13**			X				Dysthymic disorder, anxiety, OCD
**14**		X	X				Depressive symptoms, OCD, alcohol dependence, ADHD
**15**			X				Anxiety, personality disorder
**16**					X		ADHD
**17**	X				X		ADHD, obsessive-compulsive traits, conduct/impulsivity disorder
**18**			X	X			Anxiety, functional disorder
**19**			X		X		ADHD
**20**	X		X				Anxiety
**21**	X						ADHD, conduct/impulsivity disorder

The number highlighted in blue means female patient. SSRIs: selective serotonin reuptake inhibitor; TCAs: tricyclic antidepressant; ATYP: atypical antidepressant; BZD: benzodiazepine; ANTIEPI: antiepileptic; METHYL: methylphenidate; ATOMX: atomoxetine; COMORB: psychiatric comorbidity(s); OCD: obsessive-compulsive disorder; ADHD: attention deficit hyperactivity disorder; ED: eating disorder

Table S7. CGI-S at first visit according to age group and sex.

CGI-S at 1st visit	Total (n=36)	C/A (n=17)	Adults (n=19)	*p* value	Females (n=13)	Males (n=23)	*p* value
Tics:<br>- Mean (SD)<br>- Median (range, IQR)	4.1 (1.15)<br>4 (2-7, 2)	3.9 (1.09)<br>4 (2-6, 1.5)	4.3 (1.2)<br>4 (3-7, 2)	0.415	4.1 (1.14)<br>4 (3-7, 2)	4.1 (1.18)<br>4 (2-6, 2)	1
Comorbidities:<br>- Mean (SD)<br>- Median (range, IQR)	3.8 (1.57)<br>4 (1-7, 2)	3.17 (1.42)<br>3 (1-5, 2)	4.2 (1.55)<br>4 (1-7, 2)	0.042	4.5 (1.12)<br>4 (7, 1)	3.35 (1.67)<br>3 (1-6, 3)	0.75
Global:<br>- Mean (SD)<br>- Median (range, IQR)	4.0 (1.08)<br>4 (2-7, 2)	3.9 (1.08)<br>4 (2-6,1.5)	4.3 (1.2)<br>4 (3-7,2)	0.175	4.1 (1.14)<br>4 (3-7, 2)	3.9 (1.06)<br>4 (2-6, 2)	0.721

C/A: children and adolescents. 1= normal; 2= borderline ill; 3= mildly ill; 4=moderately ill; 5= markedly ill; 6= severely ill; 7= among the most extremely ill patients.

Table S8. CGI-S at last visit according to age group and sex.

CGI-S at last visit	Total (n=36)	C/A (n=17)	Adults (n=19)	*p* value	Females (n=13)	Males (n=23)	*p* value
Tics:<br>- Mean (SD)<br>- Median (range, IQR)	3.2 (1.17)<br>3 (1-6, 1.75)	2.8 (1.1)<br>3 (1-5, 1)	3.6 (1.1)<br>3 (2-6, 1)	0.038	3.5 (1.3)<br>3 (2-6, 2)	3 (1.1)<br>3 (1-5, 1)	0.281
Comorbidities:<br>- Mean (SD)<br>- Median (range, IQR)	3.5 (1.52)<br>3.5 (1-6, 1)	2.6 (1.17)<br>3 (1-4, 2.5)	4.2 (1.4)<br>4 (1-6, 3)	0.002	4 (1.2)<br>4 (2-6, 2)	3.2 (1.9)<br>3 (1-6, 2)	0.169
Global:<br>- Mean (SD)<br>- Median (range, IQR)	3.5 (1.11)<br>3 (2-6, 1)	3 (0.9)<br>3 (2-5, 1)	3.9 (1.1)<br>4 (3-6, 2)	0.010	3.8 (1.2)<br>4 (2-6, 2)	3.3 (1.0)<br>3 (2-6, 1)	0.312

C/A: children and adolescents. 1= normal; 2= borderline ill; 3= mildly ill; 4=moderately ill; 5= markedly ill; 6= severely ill; 7= among the most extremely ill patients.
